# MRI Compatible Lumbopelvic Movement Measurement System to Validate and Capture Task Performance During Neuroimaging

**DOI:** 10.1109/TNSRE.2024.3380057

**Published:** 2024-03-27

**Authors:** Ahyoung Song, Kerrigan Sunday, Sheri P. Silfies, Jennifer M. C. Vendemia

**Affiliations:** Applied Biomechanics and Movement Science Laboratory, Department of Physical Therapy, Texas State University, Round Rock, TX 78665 USA; Applied Neuromechanics Laboratory, Department of Exercise Science, University of South Carolina, Columbia, SC 29208 USA; Applied Neuromechanics Laboratory, Department of Exercise Science, University of South Carolina, Columbia, SC 29208 USA; Neurocognition and Pain Laboratory, Department of Psychology, University of South Carolina, Columbia, SC 29208 USA

**Keywords:** Biomechanics, chronic low back pain (cLBP), functional magnetic resonance imaging (fMRI), movement sensor

## Abstract

Research suggests that structural and functional changes within the brain are associated with chronic low back pain, and these cortical alterations might contribute to impaired sensorimotor control of the trunk and hips in this population. However, linking sensorimotor brain changes with altered movement of the trunk and hips during task-based neuroimaging presents significant challenges. An MRI-safe pressure measurement system was developed to ensure proper task completion during neuroimaging by capturing movement patterns of the trunk (sensors under the lower back) and hips (sensors embedded in the foam roll under the knees). Pressure changes were measured outside of the scanner by digital differential pressure sensors to capture time-series data and analog pressure gauges for real-time determination of task performance occurring within an MRI bore during brain imaging. This study examined the concurrent validity of air pressure changes between the digital and analog sensors. The digital and analog data were compared in 23 participants during the performance of modified bilateral and unilateral right and left hip bridges. Spearman’s correlations were calculated for each sensor during the three bridging tasks and showed high positive correlations, indicating that over 87% of pressure change from the analog gauge can be explained by the pressure from the digital sensor. Bland-Altman plots showed no bias and mean differences were under three mmHg. This pressure system improves the rigor of future studies by validating the digital data from the system and increasing the capabilities of capturing lumbopelvic task performance occurring inside the scanner bore.

## I. Introduction

Studies suggest that structural and functional changes within the brain are associated with chronic low back pain (cLBP) [1], [2], [3]. Recent magnetic resonance imaging (MRI) studies reveal changes in functional connectivity, the volume of gray matter, and white matter integrity in people with cLBP, specifically within brain regions such as primary sensorimotor cortices (S1/M1), secondary somatosensory area (S2), medial and dorsolateral prefrontal cortex, and anterior and posterior cingulate cortex [4], [5], [6], [7], [8]. These cortical alterations might contribute to impaired sensorimotor control in this population. Interconnection and well-organized communication in sensorimotor cortices are critical to motor planning and execution of lumbopelvic movement [9], [10] as it requires coordinated control of the multisegmented spine, pelvis, and thighs with activation of multiple muscles [11], [12] to generate a gross movement. Receiving somatosensory information from these body regions and its integration into motor output is essential to achieve coordinated movement control and stabilization of the lumbopelvic region [13], [14]. Although researchers have reported that altered sensorimotor integration might be induced by reduced neural drive in M1 or decreased proprioceptive input in persons with cLBP [15], [16], [17], there still needs to be systematic investigations into how sensorimotor integration is related to chronic pain and altered movement patterns.

Thus, our recent work provided a new approach for investigating cortical sensorimotor integration in people with cLBP using small amplitude lumbopelvic movements during functional magnetic resonance imaging (fMRI) scanning [18]. Specifically, we modified a common lumbopelvic movement/exercise (trunk/hip bridging) so that it could be performed inside the scanner bore without excessive head motion [18]. Both bilateral and unilateral voluntary movement of the lumbopelvic region, i.e., bridging (pelvic lift), were performed during fMRI scanning to understand how the sensorimotor cortical regions integrate somatosensory information during motor planning and control and how this integration differs between people with and without cLBP.

**Fig. 1.**
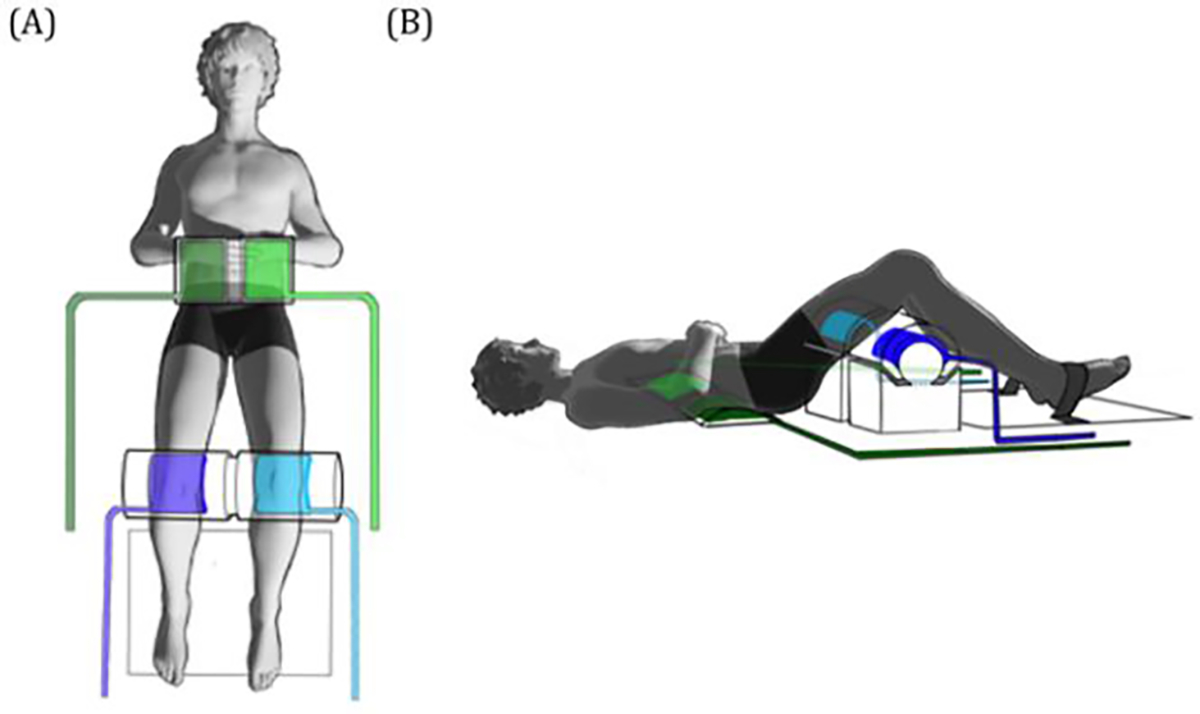
(A) Set up of participant in fMRI scanner; (B) instrumented foam roll placed under participant’s knees and air bladders placed under lower back and inflated to support individual lumbar curvature.

Because, in an MRI scanner, participants are primarily in the supine position where the trunk, pelvis, and hips are inside the bore and in contact with the scanner table, the validation of task performance and movement patterns of this region while brain scanning has been limited. To better verify task performance and capture data for further examination of the movement pattern, we developed an air pressure system instead of using other options (electromyography-EMG, photo-optic system, etc.) for the following reasons: 1) research focus, 2) access to the measurement regions, 3) system adaptability, and 4) measurement burden. First, the research focused on the link between gross lumbopelvic movement and cortical activation rather than the inference of movement patterns from synergistic models built from EMG measures of discrete muscle activations. Second, the lumbopelvic region to be monitored is inside the scanner bore and the supine position makes it hard to use a motion capture system. Third, the system needed to be adaptable to individuals with a range of anthropometric characteristics and accommodate individual variations in resting lumbar posture (see Fig 1B). Lastly, the protocol required a system with the smallest possible measurement burden during experiment set-up and as minimal burden on participants as possible. This system collects time-series digital pressure differentials and also displays analog pressure data from the bilateral lower back and knees in the scanner control room.

As a part of a larger research project and groundwork for further data analysis integrating biomechanics and brain imaging, the current study aimed to examine the concurrent validity of this instrumented lumbopelvic movement detection system during fMRI scanning. Analog sensors were used to determine the accuracy of the digital sensors integrated into the closed pressure system designed to capture participants’ task performance.

## II. Methods

### A. Participants

A total of 23 individuals, 18 with cLBP and 5 healthy controls, provided written and informed consent to participate in the study protocol approved by the University of South Carolina Institutional Review Board (IRB No. Pro00079198). The healthy control group (age: 28±7 yrs., height: 1.74±0.08 m, weight: 76.52±11.63 kg, sex: 3 females/2 males, body mass index (BMI): 25.28±2.92 kg/m^2^) met the following inclusion criteria: 1) no current health condition or musculoskeletal pain, 2) between 18–65 years of age, and 3) had no current or prior history of back pain that resulted in their seeking medical attention for their symptoms. The cLBP group (age: 35±12 yrs., height: 1.68±0.09 m, weight: 82.68±21.10 kg, sex: 12 females/6 males, BMI: 28.01±5.30 kg/m^2^) met criteria of non-specific cLBP for at least three months, pain impacting function for at least half the days per week, and age within 18–65 years. All participants had no history of spinal, abdominal, or hip surgery, inflammatory joint disease or cancer within the last five years, or medical or psychological conditions that would contraindicate MRI safety.

### B. Procedures

Participants provided demographic information and any back pain or injury history before they went through fMRI scanning. A task-based fMRI protocol was designed to assess cortical activation differences during lumbopelvic movement tasks. Participants performed a modified bilateral and unilateral right and left hip bridging in which they actively pressed their knees into a rigid foam roll and raised their lower trunk/pelvis while lying on their back in an MRI scanner (Fig 1A–B). A bilateral bridging (BB) task was performed as participants were asked to slightly lift their lower trunk/pelvis evenly by pressing their knees into the instrumented rigid foam roll (no pressure through heels) and to maintain the position for 10 seconds. Unilateral right (URB) or left bridging tasks (ULB) were performed as participants were instructed to raise their lower trunk/pelvis evenly but press only one knee into the rigid foam roll and not use the opposite side knee. Before performing the task in the fMRI scanner, participants had practice sessions in a lab and at the scanner to ensure they correctly performed the tasks while keeping their heads as still as possible and just lifting enough to clear the scanner bed [18]. The actual bridging tasks inside the scanner were performed randomly, with six repetitions for each task.

**Fig. 2.**
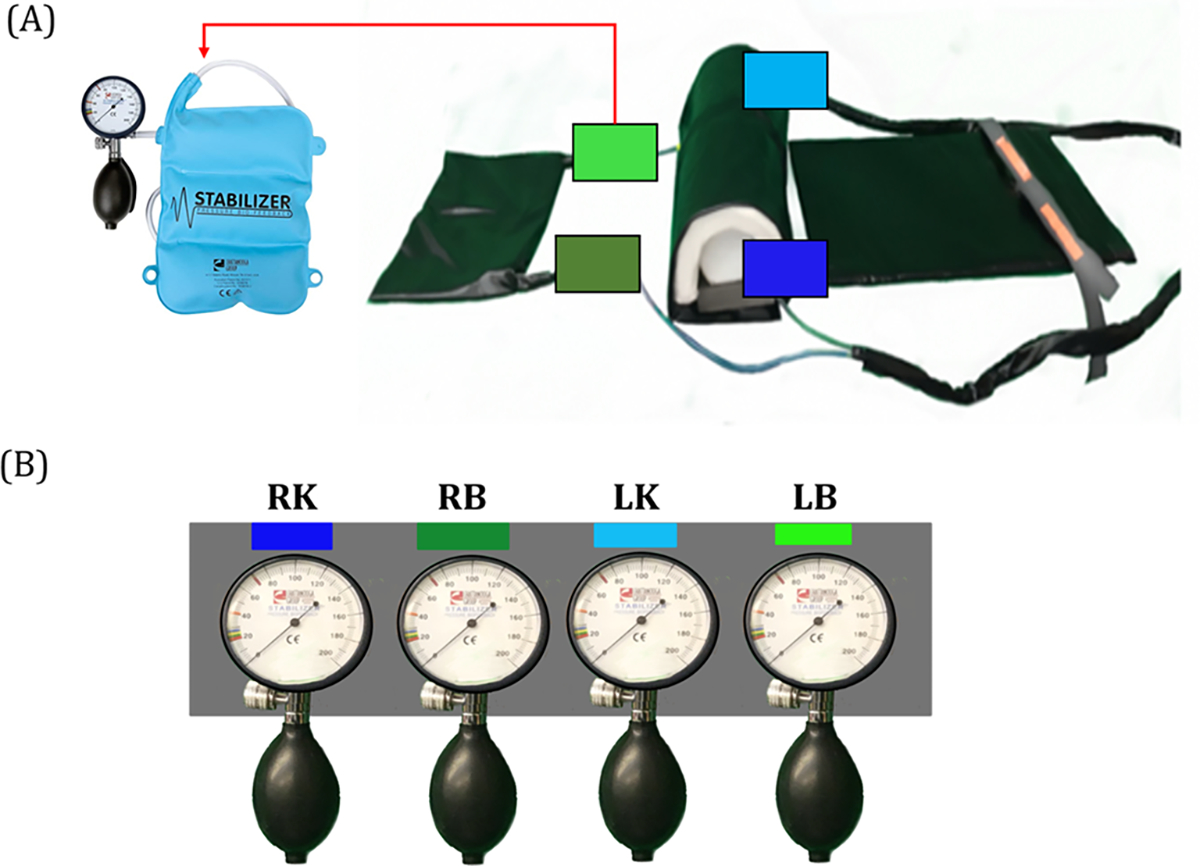
(A) The pressure measurement system adapted four commercial air bladders (Stabilizer, Chattanooga Group, USA). Two air bladders are embedded inside the rigid foam roll and two in a separate component that could be positioned under the lower trunk; (B) analog pressure gauges outside the scanner room provided real-time feedback of pressure changes and were video recorded during participant scanning for pressure data collection. RK, right knee; RB, right back; LK, left knee; LB, left back.

The MRI-safe closed pressure measurement system was developed using separate air bladders under the right and left lower trunk to capture lower trunk/pelvis motion. To capture hip motion, separate air bladders were embedded in a rigid foam roll supporting the right and left knees (Fig 2A). Four air hoses ran from the air bladders through a portal into the MRI control room. Details of the system are available at https://doi.org/10.17605/OSF.IO/ZNAU7. The four in-line analog gauges were mounted to a board in the control room. This new device needed to be able to 1) provide information about pressure changes in real-time (analog) to allow verification of the specific task and maintenance of the trunk support and position during scanning and 2) capture pressure data digitally, allowing later evaluation of the participant’s movement pattern (intersegment coordination) during lumbopelvic movement tasks.

**Fig. 3.**
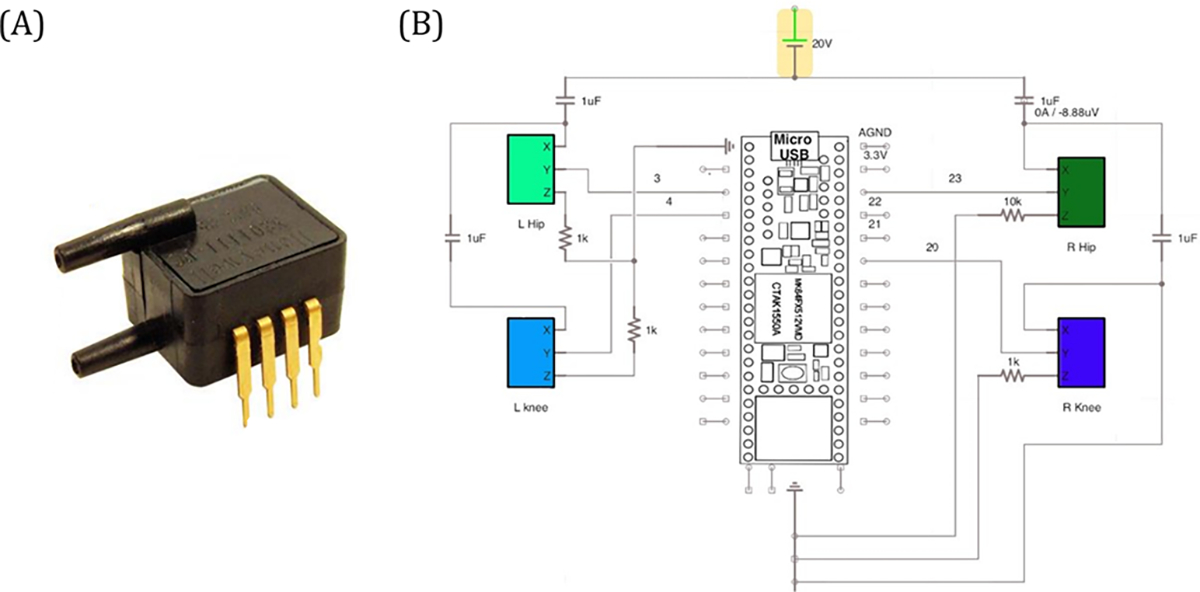
(A) A low-pressure ASDX series silicon pressure sensor; (B) the circuit for capturing the digital output from four pressure sensors.

The system was pressurized to support the participant’s natural lumbar curvature, and the knee air bladders were set at standardized pressure levels while the participant was lying relaxed on the scanner bed. Pressure changes were measured outside the scanner in line with the system by analog (Fig 2B) and digital pressure sensors (Fig 3A). Separate analog gauges represented the pressure under the right knee (RK), right back (RB), left knee (LK), and left back (LB). The dials from these pressure gauges were videotaped (sampling rate of 30Hz) for the entire experimental session. In addition, time series digital pressure data were collected with a sampling frequency of 28Hz from four Low-Pressure ASDX Series Silicon Pressure Sensors (Honeywell, USA) placed in line with the analog sensors in the closed system (Fig 3B). The digital data were collected on a separate computer synchronized with the auditory stimulus software (e-Prime, Psychological Software Tools, USA) that also controlled the MRI.

### C. Data Analysis

**Fig. 4.**
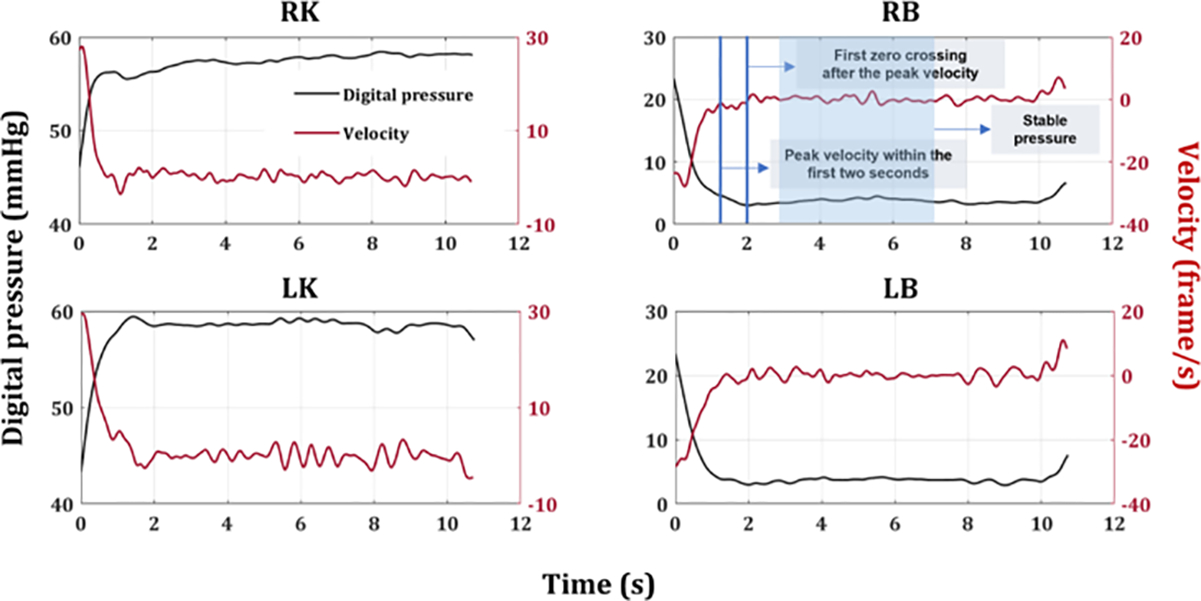
Example of participant time-series digital pressure (mmHg) from the right (top panel) and left (bottom panel) knee and back during a single BB task. A black and a red line in each graph represents filtered digital pressure and its velocity (rate of change in digital pressure), respectively. A stable maximum (or minimum) of digital pressure was determined as a 4-second averaged pressure value after a 1-second delay following the first zero-crossing point within the 2 seconds of peak velocity. For example, in the RB graph on the top right, the first blue vertical line indicates the peak velocity (maximum velocity) within the first two seconds of the task. The second blue vertical line indicates the first zero-crossing point after identifying the peak velocity. Then, the pressure was averaged for a 4-second period following a 1-second delay after the first zero-crossing point. RK, right knee; RB, right back; LK, left knee; LB, left back.

Analog pressure gauges were used to control system pressurization and independently verify the accuracy of the digital sensors. To compare the digital and analog pressure measures, the stable maximum pressure (mmHg) of knee sensors and the stable minimum pressure of back sensors were recorded during the holding phase of the bridge position. The analog pressure data were obtained from the video that a trained observer later recorded using the precision of the analog pressure gauge (2mmHg). The digital sensors provided a means to capture time-series data instead of the more limited measurements of analog pressure changes at one to two points during the task. Digital pressure data was filtered with a dual pass Butterworth filter (2Hz cutoff), and velocity (slope) was calculated in MATLAB (2022a). To find the stable maximum (knee) and minimum pressure (back), the first zero-crossing point within 2 seconds of peak velocity was identified. After a 1-second delay following the first zero-crossing, 4 seconds of pressure data were averaged to represent the stable pressure reading (Fig 4). Analog and digital stable values were averaged over the six trials for each bridging task.

During the bilateral bridging task, pressure from both back sensors (RB and LB) was expected to decrease as participants evenly raised their pelvis and back. Conversely, pressure from both knees (RK and LK) was expected to increase as they pressed down on the sensors inside the rigid foam roll using both knees to achieve hip extension. For the unilateral bridging task, pressure from back sensors was expected to decrease, similar to the bilateral bridging task, but the pressure from only one knee sensor was expected to increase as they used one knee to press into the rigid foam roll and raise their lower trunk and pelvis (e.g., only right side of knee pressure should increase during URB task and vice versa).

### D. Statistical Analysis

A priori power analysis for correlations was performed using G*Power3 [19]. We used *ρ*H_1_ = 0.9, *ρ*H_0_ = 0.7, power 0.80, and p < 0.05, which required a total sample of 20 participants. For system performance to be acceptable for future research paradigms, the correlations must be at least 0.9.

Data normality assumptions were not met. Spearman’s rho (*ρ*) correlation analyses with 95% confidence intervals were performed to determine the relationship between digital and analog pressure measures for each sensor (RK, RB, LK, and LB) during the three bridging tasks. Bland Altman plots were used to visualize the differences between the two measurement techniques, establish the limits of agreement, and identify potential bias in pressure measurements.

## III. Results

**Fig. 5.**
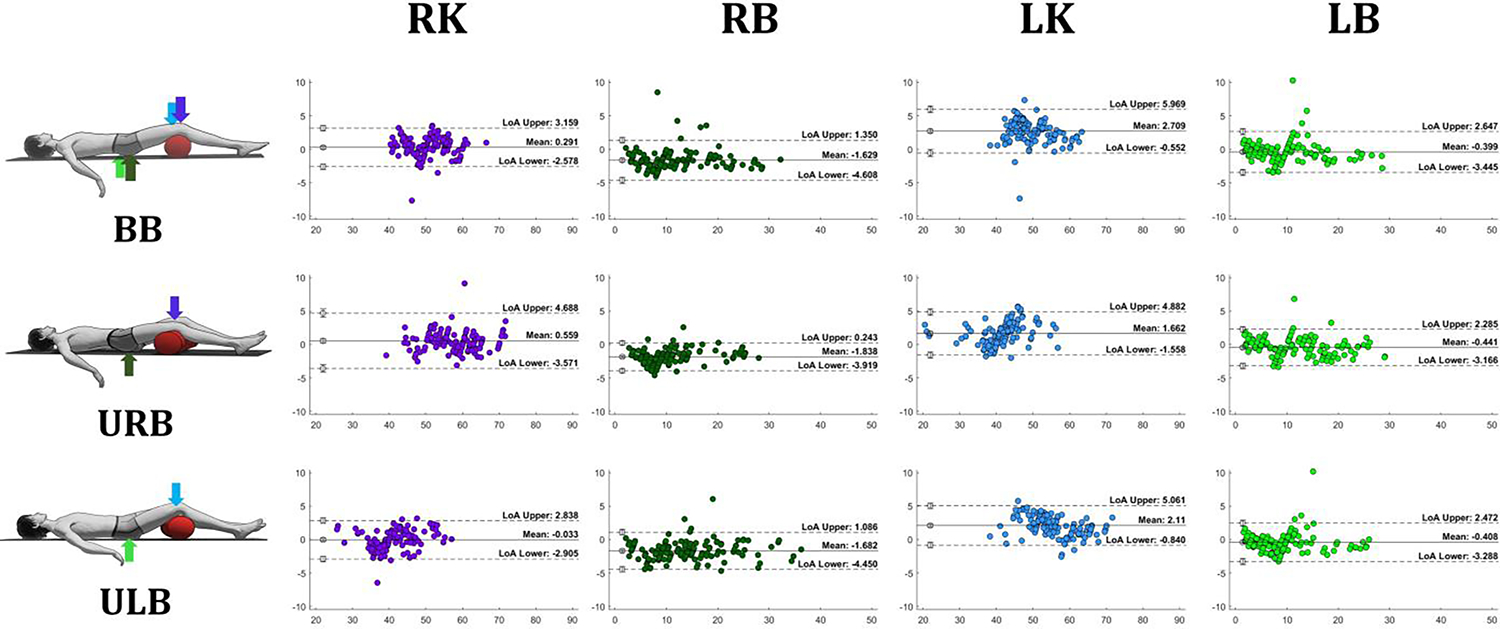
Bland-Altman plots show that the difference between digital and analog pressure is less than 3mmHg during bilateral and unilateral bridges. BB, bilateral bridging; URB, unilateral right bridging; ULB, unilateral left bridging; RK, right knee; RB, right back; LK, left knee; LB, left back.

The mean stable maximum (knee) and minimum (back) of analog and digital pressure across participants during all three bridging tasks are shown in Table I. All tasks and sensors show high positive correlations, indicating that over 87% of pressure changes (lowest *ρ* = 0.937) from the digital sensor can be explained by the pressure from the analog gauges (see Table II). In addition, Bland-Altman plots (Fig 5) did not demonstrate bias, and mean differences between these two pressure measurements were under 3mmHg across all three bridging tasks.

## IV. Discussion

This study examined the concurrent validity of pressure measurements used to monitor participants’ compliance with movement instruction during brain scanning and lumbopelvic movement patterns in people with cLBP and healthy controls. We observed high correlations and small mean differences between analog and digital pressure measurements. These findings indicate that using a closed-air pressure system and digital pressure differential sensors is feasible during task-based fMRI and that the digital pressure sensor data can be used to document task accuracy and capture the movement patterns of participants.

One of the well-documented deficits in individuals with cLBP is diminished postural and trunk movement control, which is critical to performing daily functional activities [20]. For example, a bridging task requires controlling the weight of the lower trunk while maintaining body balance and position by generating forces through trunk and hip muscle activations [21]. In addition, maintaining dynamic spine stability during this position involves coordinated contraction of global and local muscles by accurately integrating sensory information from the region, central processing, and proper motor output [22], [23].

Our bridging task would be a difficult task to capture and monitor given that it occurs within the MRI bore itself, unlike motor tasks outside the scanner, such as pedaling [24], stepping [25], or ankle flexion [26], [27] with lower limbs. Researchers have attempted to link changes in brain activation obtained from fMRI to muscle activity and movement during brain scanning by applying different approaches. Previous literature has used visual inspection for movement verification during fMRI scanning, such as finger tapping and ankle movement in individuals with stroke [28], [29]. Other researchers used more advanced kinematic analysis methods, such as a motion capture system installed inside the MRI room, to collect and analyze ankle motion outside the scanner bore [26]. Surface EMG on hand, ankle, and wrist muscles has been used to quantify distal isometric activity, and the utility of EMG and event-related fMRI for detecting the onset and offset of action has been demonstrated [30], [31]. However, the supine position in the MRI scanner does not easily allow the proximal attachment of surface EMG electrodes of thoracic and lumbar musculatures or reflective markers for a motion capture system. MRI-compatible force sensors have also been used to detect the force level of both feet applied during simulated gait initiation (step initiation) in elderly participants with Parkinson’s disease [25]. Again, while these tools have been used to assess participants’ compliance with the requested task and quantify aspects of movement control, they were primarily focused on the movement of peripheral body segments, such as the hand and fingers, foot (ankle joint), or shank and thigh (knee joint), where a majority of movements were performed outside of the MRI bore.

When placed under a participant, the developed air pressure system supported their lower spine curvature and allowed the capture of lumbopelvic movement patterns during the bridging task. Unlike employing observation of task compliance in the scanner or mean muscle activations in individual muscles using surface EMG, the air pressure system could provide a comprehensive understanding of how people with and without cLBP control and move their lumbopelvic region during task performance. Real-time monitoring through this system also allows the researcher to correct participants who are not doing the task appropriately, thus improving data quality. Furthermore, it does not require modification of an inherent feature of fMRI in that participants can comfortably lie down on their back without changing body position to be measured.

## V. Conclusion

The development of this air-pressurized system improves the rigor of the larger study by validating the digital system and increasing the monitoring capabilities of lumbopelvic movements occurring in the scanner bore. The time-series digital data provide an opportunity to assess the participant’s movement patterns. Validation of the system allows us to move beyond task-based verification to movement pattern analysis. In addition, it opens the possibility of adapting the approach for monitoring tasks or movement patterns of other body regions enclosed with the bore during neuroimaging.

## Figures and Tables

**TABLE I:** Mean (mmHg, +std) Analog and Digital Pressure From Each Sensor During BB, URB, and ULB Tasks

		RKA	RKD	RBA	RBD	LKA	LKD	LBA	LBD

BB	Mean	50.55	50.84	12.23	10.60	48.07	50.77	9.70	9.30
	Std	5.39	5.36	6.61	6.64	5.37	5.05	6.33	6.14
URB	Mean	57.43	57.99	11.30	9.46	40.52	42.18	12.10	11.66
	Std	6.54	6.65	5.51	5.76	5.91	6.36	6.76	6.52
ULB	Mean	41.02	40.99	14.62	12.94	54.43	56.54	9.22	8.82
	Std	5.30	5.72	7.57	7.70	6.80	6.17	6.01	5.97

BB, bilateral bridging; URB, unilateral right bridging; ULB, unilateral left bridging; RKA, right knee analog; RKD, right knee digital; RBA, right back analog; RBD, right back digital; LKA, left knee analog; LKD, left knee digital; LBA, left back analog; LBD, left back digital.

**TABLE II:** Spearman’s Rho (*ρ*) Correlations Between Digital and Analog Pressure for Each Sensor Across the Three Tasks

Task	Sensor	Correlation Coefficient (*ρ*) (95% CI [LL, UL])

BB	RK	.970 * [.958, .979]
	RB	.962 * [.946, .973]
	LK	.937 * [.912, .965]
	LB	.969 * [.956, .978]
URB	RK	.945 * [.923, .961]
	RB	.974 * [.963, .981]
	LK	.962 * [.947, .973]
	LB	.978 * [.969, .984]
ULB	RK	.968 * [.954, .977]
	RB	.983 * [.976, .988]
	LK	.983 * [.976, .988]
	LB	.974 * [.964, .982]

* *p* < .001, BB, bilateral bridging; URB, unilateral right bridging; ULB, unilateral left bridging; RK, right knee; RB, right back; LK, left knee; LB, left back; CI, confidence Interval; LL, lower limit; UL, upper limit.
